# Fibril-mediated oligomerization of pilin-derived protein nanotubes

**DOI:** 10.1186/1477-3155-11-24

**Published:** 2013-07-05

**Authors:** Anna Petrov, Stephanie Lombardo, Gerald F Audette

**Affiliations:** 1 Department of Chemistry, York University, Toronto, ON M3J1P3, Canada; 2 Centre for Research on Biomolecular Interactions, York University, Toronto, Canada

**Keywords:** Protein nanotubes, Type IV pilin, Type IV pilus, Protein oligomerization

## Abstract

**Background:**

Self-assembling protein nanotubes (PNTs) are an intriguing alternative to carbon nanotubes for applications in bionanotechnology, in part due to greater inherent biocompatibility. The type IV pilus of the gram negative bacteria *Pseudomonas aeruginosa* is a protein-based fibre composed of a single subunit, the type IV pilin. Engineered pilin monomers from *P. aeruginosa* strain K122-4 (ΔK122) have been shown to oligomerize into PNTs both in solution and at surfaces. In order to fully exploit PNTs in bionanotechonological settings, an in-depth understanding of their assembly, physical characteristics and robustness, both in solution and when constrained to surfaces, is required.

**Results:**

This study details the effectiveness of multiple initiators of ΔK122-derived PNT oligomerization and characterize the formation of PNTs in solution. The optimal initiator for the oligomerization of ΔK122 in solution was observed to be 2-methyl-2,4-pentanediol (MPD). Conversely, larger PEG molecules do not trigger oligomerization. Multi-angle light scattering analysis indicates that the pilin protein exists in a monomer-dimer equilibrium in solution, and that an intermediate species forms within three hours that then coalesces over time into high molecular weight PNTs. Transmission Electron Microscopic analysis was used to observe the formation of oligomerized ΔK122 fibrils prior to assembly into full-length PNTs.

**Conclusions:**

The oligomerization of ΔK122 pilin derived PNTs is a fibril mediated process. The optimal trigger for PNT oligomerization in solution is MPD, and the observation that PEGs do not induce oligomerization may enable the oligomerization of pilin-derived PNTs on PEG-functionalized surfaces for implantable bionanodevices.

## Background

The development of peptide and protein-based nanotubes as biologically accepted nanosystems have several advantages when compared to their inorganic counterparts such as carbon nanotubes (CNTs), which are significantly more cytotoxic and present biocompatibility issues [1-11]. Peptide and protein based nanotubes can be assembled utilizing both template and non-template assembly mechanisms under milder conditions (ambient temperature, physiological pH), and provide a readily customizable system via modern protein engineering methods [12,13]. In addition, studies have shown that wormlike, filamentous nanoparticles are better than spherical ones at avoiding immune responses allowing for longer circulation times due to the difficulty of macrophages have adjusting tertiary and/or quaternary structure to engulf such elongated particles [14-16]. Therefore, peptide and protein-based nanotubes will likely have applications as drug-delivery vehicles as their relatively large inner cavity and high surface areas would enable them to transport drug molecules, nucleic acids or antigens to targeted cell surface.

Several recent studies have examined the applicability of nanotubes from peptides [17-20], proteins [21-29], and viruses [30-38]. For instance, the mutation of the pIII and pVII coat proteins of the M13 phage enabled the modified phage to scaffold metal oxides [34,35]. The resultant protein-metal hybrid bionanowires demonstrated significant initial and reversible storage capacity [35,38], suggesting the utility of these nanocomposites for power generating applications. Another system ex-amined for protein nanotube (PNT) development is based on the bacterial flagella, where the flagellin protein FliC has been modified to contain a thioredoxin domain [21]. The resultant FliC-thioredoxin chimera was shown to form PNTs on surfaces [22] as well as enable metal nanowire synthesis [39]. These studies highlight the applicability of using bio-inspired PNTs for various applications in nanoelectronics and as biosensors.

In addition to protein-based nanostructures derived from viral coat proteins and flagella, PNTs have been shown to assemble from an engineered form of the type IV pilin [23,24], the monomeric unit of the type IV pilus (T4P) of many gram-negative bacteria including *Pseudomonas aeruginosa*. Opportunistic infections by *P. aeruginosa* are a significant cause morbidity and mortality in individuals with compromised immune systems (*e.g.* burn victims [40] and cystic fibrosis patients [41]), with infections being initiated through interaction of T4P with cellular receptors [42-47]. In addition to cellular adherence, T4P are involved in a number of functions including surface adherence [48,49], twitching motility [47,50-54], DNA uptake [55-57], and biofilm formation [47,58-60]. T4P are robust structures assembled and disassembled via a membrane-spanning complex whose architecture is evolutionarily related to a type II secretion system [47,50,60]. *P. aeruginosa* T4P have also been demonstrated to retract at rates of 0.5-1 μm s^-1^ (~1500 subunits s^-1^) [51] generating forces exceeding 100 pN [61]. The T4P has an outer diameter of approximately 6–8 nm and can reach lengths up to tens of microns [44,46,47,50,62-65]. T4P are polymers of the type IV pilin, and cryo-EM [66-68] and fibre diffraction [69] analyses of T4P have demonstrated that T4P exhibit a three-start helical assembly of pilin monomers [44,62]. The type IV pilin monomer is comprised of a four-stranded antiparallel β-sheet wrapped around a hydrophobic α-helix connected by a variable loop region [66,70-76]. Surface adherence and cell-host adhesion is mediated by a C-terminal loop known as the D-region, which is disulfide-bound in most pilins [66,68,70-74,76], although the FimA pilin of *Dichelobacter nodosus* displays a conserved structure without the disulfide bond [75]. The observation that truncated pilins from *P. aeruginosa* strain K122-4 (ΔK122) could form PNTs morphologically similar to T4P in the presence of a hydrophobe (C_11_-SH), both in solution and when the hydrophobe was surface constrained [23,24], presents an interesting avenue for the development of bionano applications that target the T4P, for example pilus-specific biosensors.

Several studies highlight the potential applications of PNTs including targeted drug delivery systems, tissue-engineering scaffolds and biosensing devices [35,38,77-83]. However, reports characterizing the assembly and properties of PNTs generated from full-length proteins in solution or at surfaces are more limited; it is in this light that we undertook the characterization of the oligomerization of pilin-derived PNTs in solution. Pilin-derived PNTs may have an advantage of being a more biologically accepted nanosystem when compared to their CNT counterparts. However in order to fully exploit PNTs for application development, a detailed understanding of their assembly and physical characteristics in solution and when surface-constrained is required. In the current study, we examine the assembly of ΔK122-derived PNTs in solution, monitoring PNT oligomerization through liquid chromatography, multi-angle light scattering and negatively stained transmission electron microscopic methods. We identify an optimal trigger molecule, 2-methyl-2,4-pentanediol (MPD), characterize pilin oligomerization in solution, and discuss the assembly of ΔK122-derived PNTs through intermediate pilin fibrils.

## Results and discussion

The identification that monomeric pilins from *P. aeru-ginosa* oligomerized into PNTs [12,13,23] suggests that these structures could be adapted for a variety of applications. Previous studies, employing a polyclonal antibody that recognizes the C-terminal region of the pilin from multiple strains of *P. aeruginosa*[84-86], have shown that the structure of and receptor binding properties of ΔK122 are unaffected upon oligomerization into PNTs [23,48,55]. Furthermore, the observation that pilin-derived PNTs can assemble both in solution [23] and at surfaces [24,49] suggests that these structures could be adapted for applications such as biosensors and in bionanoelectronics while retaining several functional features associated with the native pilus itself.

**Figure 1:**
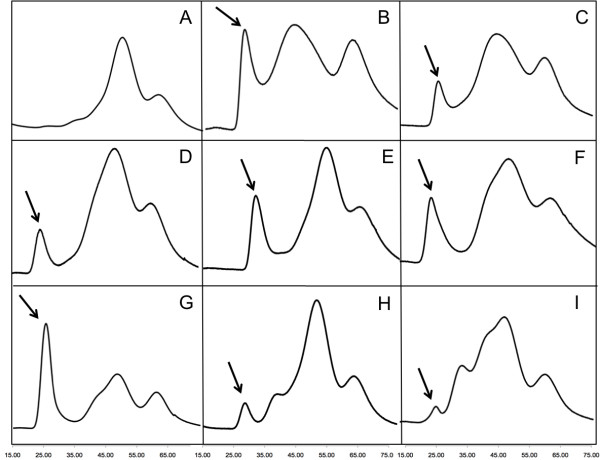
**The effect of hydrophobic trigger molecules on PNT oligomerization in solution.** SEC analysis (Sephadex G50 resin) of 15 mg·mL^-1^ ΔK122 incubated for 96 hours at room temperature with the addition of: **(A)** buffer only (10 mM Tris, 300 mM NaCl, 1 mM EDTA, 1 mM DTT, pH 7.4; no additives); **(B)** the hydrophobic trigger solution as reported by Audette *et al*. (1-undecanethiol, 1-propanol, methanol, DTT, EDTA, pH 6.4) [23]; panels **C** through I are buffer with the addition of 1-undecanethiol **(C)**, 1-tetradecanethiol **(D)**, methanol **(E)**, 1-propanol **(F)**, 2-methyl-2,4-pentanediol **(G)** and PEG 3350 **(H)** or PEG 8000 **(I)**. All chromatograms are on a common vertical scale, and the effectiveness of each compound to initiate pilin oligomerization was evaluated by the appearance of the high molecular weight peak indicated with an arrow in each panel versus the concurrent decrease in the peak associated with the ΔK122 monomer **(**panel **A)**.

Initial studies of pilin-derived PNTs in solution demonstrated that PNTs could be formed in the presence of long chain alkylthiols [23], and are stable in various aqueous buffers [23,48,49,55]. Surface studies have further demonstrated that longer chain alkanes are required for PNT oligomerization, although the hydrophobe is not incorporated into the PNT itself [23,24,49,87]. However, initial evidence suggested that smaller alkyl-chains could induce PNT oligomerization (GFA, unpublished observations). In order to assess the minimal hydrophobe required to initiate PNT oligomerization, freshly purified ΔK122 (15 mg·mL^-1^) was incubated with a series of trigger molecules for 96 hours and analyzed using size exclusion chromatography (SEC). Each trigger molecule's ability to initiate PNT oligomerization was interrogated via the presence and height of the void volume peak relative to that of the ΔK122 peak (Figure 1). PNT oligomerization is triggered by the "hydrophobic" component of the solution and not due to exposure to polypropylene of the microcentrifuge tubes during incubation or by buffer components themselves. When incubated in buffer alone, no high molecular weight protein peak is observed (Figure 1A). Comparatively, when incubated with the original "hydrophobe solution", a significant PNT peak is observed (Figure 1B). As expected, when incubated with either 1-undecanethiol (Figure 1C) or 1-tetradecanethiol (Figure 1D) alone in buffer, PNT oligomerization is observed. However, in both cases, PNT oligomerization is less than that observed when comparing to the original hydrophobe solution (Figure 1B). This is likely due to the low solubility of both C_11_-SH and C_14_-SH. The addition of 1-propanol and methanol in the original PNT trigger solution greatly increased the aqueous solubilisation of the alkylthiol allowing for increased interaction with the protein, which in turn increased PNT formation. Interestingly, both methanol (Figure 1E) and 1-propanol (Figure 1F) alone in buffer were able to trigger PNT oligomerization. In both cases, the PNT peaks are larger than those seen with either C_11_-SH or C_14_-SH, again indicating that solubility of the hydrophobic trigger molecule in the aqueous buffer is critical for initiation of PNT oligomerization.

The most effective trigger compound for the oligomerization of ΔK122 PNTs was found to be 2-methyl-2,4-pentanediol (MPD) (Figure 1G). MPD had been expected to behave similarly to 1-propanol in its ability to trigger PNT oligomerization. This is because the presence of the second hydroxyl group in MPD increases its hydrophilicity despite containing 2 more methylene groups than propanol. This increased hydrophilicity enables MPD to more effectively interact with proteins in solution and is therefore often used in protein crystallization [88]. In fact, the PNT peak associated with the incubation of ΔK122 with MPD is larger than any of the other samples, both in peak height as well as its ratio to the monomeric ΔK122-4 peak (Figure 1). This indicates that the added solubility due to the second hydroxyl of MPD adds sufficient hydrophilicity for a more favorable interaction with the ΔK122 monomer in order to initiate PNT oligomerization. In contrast to MPD, exposure of the protein to polyethylene glycol (PEG) 3350 (Figure 1H) or PEG 8000 (Figure 1I) shows very little PNT oligomerization. PEGs are also frequently used in protein crystallization [88,89], and have been shown to reduce non-specific adsorption of proteins to implantable devices [90]. These data are important for the future development of PNT-containing nanodevices where the bio-nonfouling nature of PEG additives as surface coatings is exploited for increasing implantable device lifetimes in the body [91]. The lack of PNT oligomerization with PEGs may allow the pre-functionalization of surfaces with PEGs and exposed hydrophobes for site-localized PNT oligomerization; we are currently examining this possibility.

**Figure 2:**
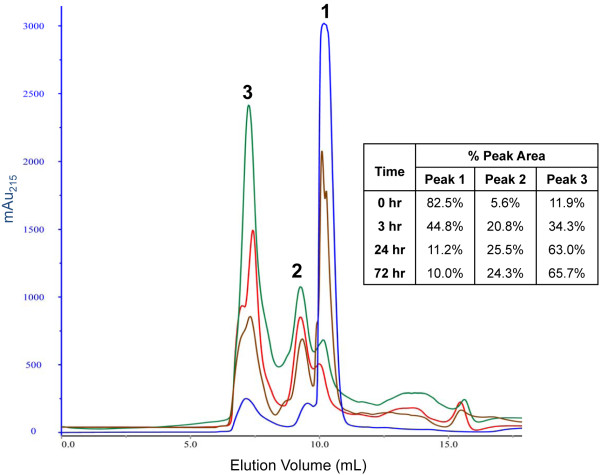
**Monitoring ΔK122 PNT generation over time.** SEC analysis (10 nm pore size) of 1 mg·mL^-1^ ΔK122 (blue) incubated with MPD in oligomerization buffer for 3 hours (brown), 24 hours (red) or 72 hours (green). The peak associated with ΔK122 (Peak 1) decreased over time with the appearance and increase in height of peaks associated with higher molecular weight species (Peaks 2 and 3). The relative areas of these peaks were integrated (inset table) to assess PNT oligomerization over time. The peaks were further characterized by MALS and TEM (Figure 3; Table 1) and identified as pilin-derived nanofibrils (Peak 2) and PNTs (Peak 3).

Previous studies of ΔK122 PNT formation [23,87] showed the appearance of a peak with a retention time longer than that of the monomeric pilin. A peak with a longer retention time was also observed when studying the optimal trigger molecule for PNT oligomerization (Figure 1). It has been suggested that this longer retention SEC peak was a result of a ΔK122 pilin fibril that interacted abnormally with the chromatographic resin [23]. The presence of a pilin fibril would be consistent with both initial TEM studies of PNTs, where the PNTs were observed to "fray" [23], as well as current three-start T4P assembly models [44,62,67]. In order to clarify the oligomerization process, and identify this predicted pilin fibril, we monitored the oligomerization of ΔK122 in solution over time on a high-resolution silica-based SEC column (Figure 2). Starting with the initial ΔK122 solution (1 mg·mL^-1^), incubation with the MPD initiator resulted in a decrease in the peak height associated with the ΔK122 monomer, with a concurrent increase in peak heights of peaks associated with higher molecular weight species (Figure 2, Peak 1 vs. Peaks 2 & 3). The presence of higher molecular weight species (Peaks 2 and 3) can be observed in as early as 3 hours (brown trace). Comparison of relative peak areas indicates that at 3 hours incubation, the monomeric form of ΔK122 decreases from 82.5% to 44.8% total species present, while the higher molecular weight species increase from 17.5% to 55.1% (Figure 2, inset table). The higher molecular weight species continue to accumulate over 24 (red trace) and 72 (green trace) hours, resulting in the higher molecular weight species accounting for 90% of the total protein present in solution after 72 hours. Of the resulting overall 90% total protein, an average of 23.5% remains in the middle range (Peak 2), suggesting that the process of PNT oligomerization is likely dependent upon the initial formation of an intermediate species (Peak 2) that then form PNTs (Peak 3) over time. It was also noted that the peak corresponding to the ΔK122 monomer (Peak 1) does not fully disappear over the process of PNT formation, remaining at ~10% of the protein present after 72 hours incubation. A possible explanation for this is that there is an equilibrium between the ΔK122 and nascent fibrils prior to incorporation into full PNTs.

**Figure 3:**
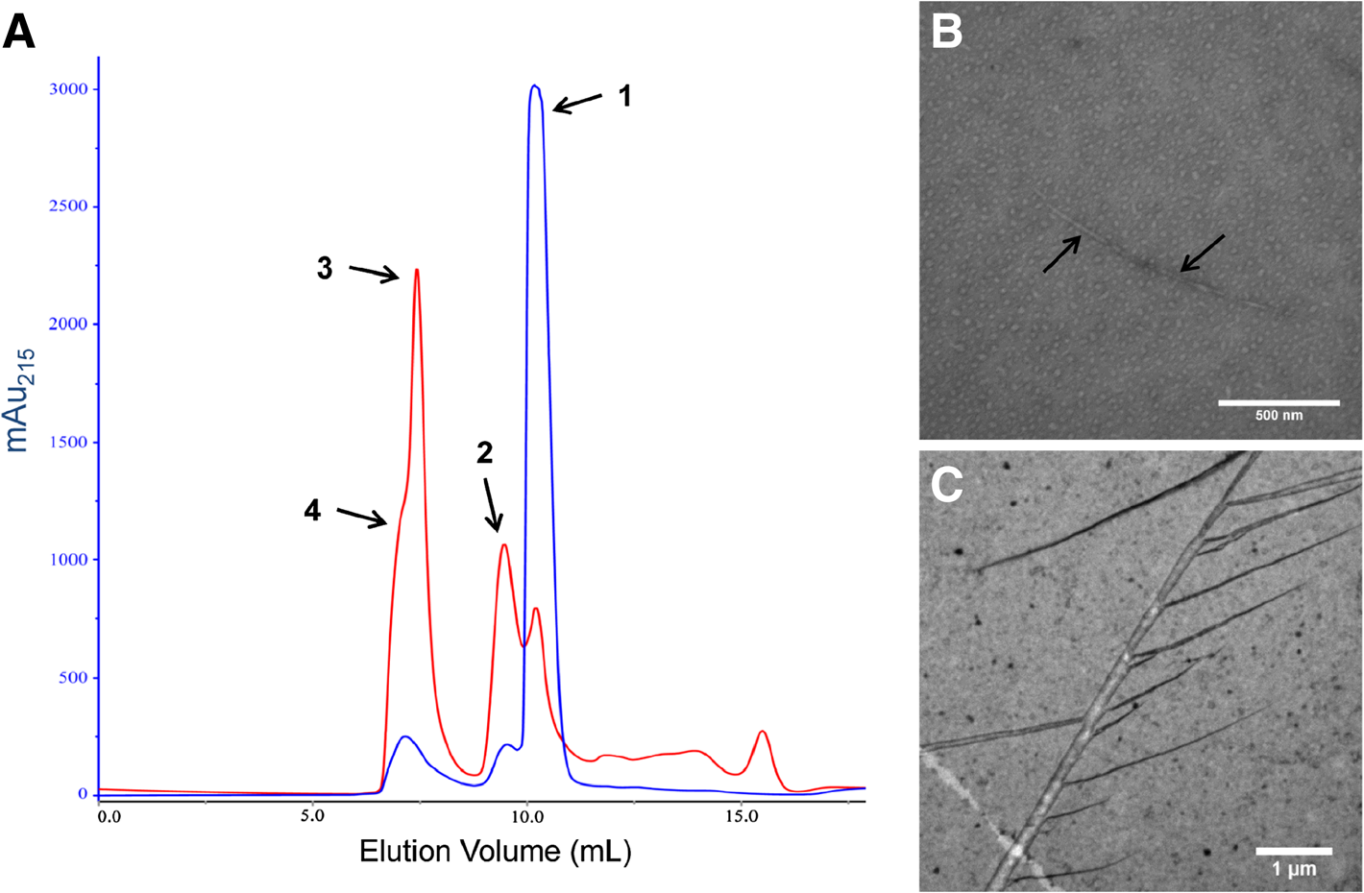
**Characterizing the process of ΔK122 PNT oligomerization in solution. (A)** SEC analysis (10 nm pore size) of 1 mg·mL^-1^ ΔK122 before (blue) and after (red) 24 hr incubation with MPD. Peaks 1 through 4 were analyzed with multi-angle light scattering in-line with the SEC (SEC-MALS; Table 1). SEC-MALS data indicate that ΔK122 exists in a monomer/dimer equilibrium (Peak 1) and that upon incubation with MPD, higher molecular weight species form, corresponding to 14, 20 and 40 pilin monomers for Peaks 2, 3 and 4, respectively (Table 1). Aliquots of SEC-separated ΔK122 (15 mg·mL^-1^) incubated with MPD for 24 hr corresponding to Peaks 1/2 and 3/4 were negatively stained with 4% uranyl acetate and visualized with TEM. **(B)** The ΔK122 monomer/dimers in Peaks 1/2 are seen as aggregates in TEM, and form pilin fibrils (highlighted by arrows) upon addition of the oligomerization initiator MPD to the protein solution. **(C)** Pilin fibrils associate into PNTs (Peaks 3/4), which can then coalesce into larger PNT bundles that show similar structures as those formed when C_11_-SH is used as the inducer of pilin oligomerization [23,87].

At incubation times up to 24 hours, the presence of a shoulder on the high molecular weight SEC peak (Figure 2; red trace) was noted, suggesting multiple high molecular weight species were present in solution. To assess the species present in solution during the assembly of PNTs from monomeric ΔK122, SEC separated samples were subjected to multi-angle light scattering (MALS) analysis following SEC separation (Figure 3A). The protein species’ weight-averaged molar mass (M_w_) and other relevant MALS data are presented in Table 1. Multiple molar mass values are reported from MALS experiments, including the number-averaged (M_n_) and weight-averaged (M_w_) molar mass; the ratio of M_w_/M_n_ (polydispersity index) is an indication of the heterogeneity of the species being analyzed [92,93]. When M_w_/M_n_ differs from unity, the species is a more polydisperse mixture and M_W_ is a generally more reliable assessment of the molecular mass of the species present. It can be seen from Table 1 that the all peaks analyzed (Figure 3A) show M_w_/M_n_ ratios indicating the presence of multiple species in each peak. Analysis of the peak corresponding to "monomeric" ΔK122 shows a MALS-determined M_w_ of 26.5 ± 1.2 kDa and hydrodynamic radius (R_h_) of 4.32 ± 0.11 nm. While the R_h_ of the pilin correlates with that known from the crystallographic structures of the ΔK122 pilin [71], the MALS-determined M_w_ was unexpected. Taking into consideration that the known molecular weight of ΔK122 is 12,837.57 Da [94], and that both crystallographically determined structures of ΔK122 have dimers with their asymmetric unit [71], these data indicate that the ΔK122 pilin exists in a monomer-dimer equilibrium prior to initiation of PNT oligomerization. In addition, electro-spray mass spectrometric analysis of ΔK122 also suggests a mono-mer-dimer equilibrium and that pilin dimers come together to form larger species after addition of the MPD trigger (D. Yong *et al*., in preparation). Furthermore, noting the generally low solubility of full-length pilins due to the presence of the hydrophobic N-terminal α-helix [66,70,75], and that long-standing solutions of ΔK122 appear to form gels in microfuge tubes (data not shown), the observation of a monomer-dimer equilibrium of ΔK122 in solution is not unexpected. SEC-MALS analysis of ΔK122 following 24 hours incubation with MPD shows several higher molecular weight species present in solution (Figure 3A, Peaks 2–4; Table 1). The protein species within these peaks were relatively mobile, with translational diffusion (D_t_) coefficients and R_h_ values similar to that observed for the ΔK122 monomer/dimer (Table 1; Peak 1), however the Mw determined for each peak suggests that these species are composed of increasing amounts of ΔK122. With an observed M_w_ of 183.6 ± 17.4 kDa, the species within Peak 2 (Figure 3A) would contain fourteen ΔK122 monomers; the species in Peaks 3 and 4 would be composed of approximately twenty and forty monomers, respectively.

**Table 1:** SEC-MALS analysis of ΔK122 oligomerization

	Peak 1^*^	**Peak 2**	**Peak 3**	**Peak 4**
**M**_**w **_**(kDa)**	26.5 ± 1.2	183.6 ± 17.4	256.7 ± 37.7	520.7 ± 146.3
**M**_**w**_**/M**_**n**_	1.11 ± 0.07	1.27 ± 0.22	1.71 ± 0.46	1.16 ± 0.49
**R**_**h **_**(nm)**	4.32 ± 0.11	4.20 ± 0.13	3.9 ± 0.1	4.1 ± 0.1
**Dt (x10**^**-9 **^**cm**^**2**^**s**^**-1**^**)**	5.82 ± 0.15	6.10 ± 0.22	6.6 ± 0.2	6.15 ± 0.2

*Peaks analyzed as indicated in Figure 3A.

In order to observe the species identified by SEC-MALS analysis, aliquots of SEC-separated ΔK122 pilin (15 mg·mL^-1^) after 24 hours incubation with MPD corresponding to Peaks 1/2 and 3/4 were negatively stained with 4% uranyl acetate, visualized with TEM (Figure 3B, C), and compared to that PNTs oligomerized using the original C_11_-SH hydrophobe as the inducer [23,87]. TEM analysis of a Peak 1/2 aliquot (Figure 3B) show pilin fibrils (highlighted with arrows) interspersed among a general aggregation of the ΔK122 monomers/dimers. The components of a Peak 3/4 aliquot shows the presence of full PNTs (Figure 3C), consistent with previous observations of pilin-derived PNTs in solution [23] and at surfaces [24]. MALS analysis of this Peak 3/4 aliquot was challenging due to the high protein concentration (15 mg·mL^-1^) resulting in signal overload at the detector. However a MALS-determined M_w_ of 51,610 ± 4,900 kDa was observed for the species in this SEC-separated aliquot. This M_w_ corresponds to a structure that is microns in length containing ~4040 ΔK122 monomers, a structure which is observed in the TEM analysis of the Peak 3/4 aliquot (Figure 3C). Pilin-derived PNTs were also observed to further bundle into larger structures where PNT bundles ranging in width from ~25-65 nm (Figure 3C) to greater than 250 nm in cross-section [95]. Given the predicted outer diameter of ~6 nm for native T4P [44,46,62,63,66,67,69] and/or K122-derived PNTs [12,23,24], the observed structures would correspond to bundles of ~4-11 PNTs.

**Figure 4:**
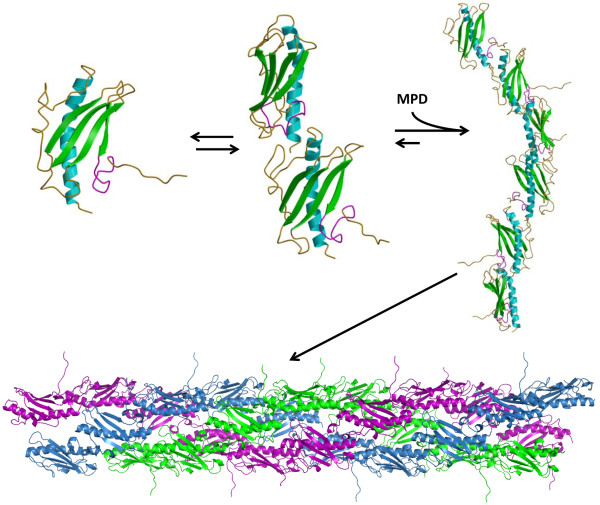
**A model of fibril-mediated pilin-derived PNT assembly.** The ΔK122 pilin (PDB ID 1QVE) [71] exists in a monomer-dimer equilibrium in solution. Upon addition of a hydrophobic initiator such as MPD to the protein solution, the ΔK122 pilin forms fibrils that can then assemble into PNTs through the coalescence of three fibrils in a three-start helical assembly as observed in native T4P [67]. Once formed, PNTs are able to form bundles (Figure 3C) and retain binding characteristics similar to native T4P [47,50,58-60]. The common structural features of the type IV pilins are highlighted on the ΔK122 monomer the 4-stranded antiparallel β-sheet (green) that is packed onto a (truncated) N-terminal α-helix (cyan) and connected by variable loop regions (gold); receptor and surface interactions by the pilins are mediated by the C-terminal D-region (magenta). The three ΔK122 fibrils that comprise the full pilin-derived PNT are shown in purple, green and blue, respectively.

T4P assembly/disassembly is achieved via a multi-protein membrane associated complex in a highly coordinated fashion [46,47,50,60,62,64,65,67]. However PNTs derived from truncated ΔK122 monomers do not have such a protein system to guide PNT assembly, nor do they have the conserved N-terminal region of the α-helix to hydrophobically drive pilin oligomerization. It is therefore not surprising that multiple species (monomers, dimers, multimers) are present in solution during PNT oligomerization. Indeed, SEC-MALS analysis of the MPD-initiated ΔK122 PNT formation (Table 1) shows that multiple species are present in solution. These multiple species are difficult to separate chromatographically as they reach higher molecular weights, however SEC-MALS data suggests that the species are likely multiples of dimers (Table 1). In addition, the observation of an initial ΔK122 monomer/dimer in solution (Figure 3A, B; Table 1) is not unprecedented knowing that both crystallographic structures of ΔK122 show two pilin molecules within their respective asymmetric units [71]. Upon addition of the MPD trigger to the protein, ΔK122 monomer/dimers would oligomerize into extended fibrils, which then can come together to form PNTs (Figure 4). If the ΔK122 PNTs oligomerized via a one-start mechanism as was initially suggested [23], one would expect ring-like structures when analyzing the contents of the intermediate molecular weight species (Figure 2, Peak 2). However a more extended structure, rather than protein rings, was observed (Figure 3B) for the intermediate pilin fibrils. When the pilin fibrils are of sufficient length, they then coalesce into PNTs that have the characteristic three-start helical symmetry seen in native T4P (Figure 4) while retaining the native structure and binding characteristics of pilins within the T4P [23,48,55]. The exact mechanism of this fibril coalescence is at this time unclear, however it may be possible that stabilization of the truncated pilin dimer and fibril is achieved through involvement of the α-helix. A comparison of the crystal [71] and NMR [73] structures of ΔK122 revealed that the α-helix of the pilin was less tightly packed on the β-sheet of the pilin in the NMR structure [71], suggesting some flexibility prior T4P assembly. Noting that there are some arguments in favour of a less rigid packing of the β-sheet onto the α-helix in the T4P [96], it is possible that the truncated α-helix of ΔK122 shifts to increase helix-helix interactions in the dimer/fibril and thereby impart increased stability to the nascent fibril prior to coalescence into PNTs. Furthermore, as native T4P are known to bundle on surfaces for coordinated motion and biofilm formation [47,50,58-60], the observation that pilin-derived PNTs also further associate and form larger bundle-like structures (Figure 3C) was expected. Research in our lab is ongoing to fully elucidate the mechanisms by which the ΔK122 pilin stabilizes the nascent fibril and to form PNTs, as well as determine the parameters of PNT bundle formation and identify how one may isolate a single PNT from a formed bundle either in solution or at surfaces.

## Conclusions

The development of protein-based nanotubes for biologically based nanosystems is receiving increased interest due to their richness in structural diversity, adaptability through protein engineering approaches and inherent biocompatibility. The adaptation of the T4P as protein nanotubes through engineered type IV pilin monomers has shown distinct promise in that these structures can assemble both in solution and at surfaces in a template independent fashion [23,24,49,87,95]. In the current report, we have shown that the ΔK122 pilin is in a monomer-dimer equilibrium in solution, and that oligomerization of the pilin can be induced from short alkyl chains in solution, however optimal oligomerization is achieved when MPD is used as an initiator (Figure 1). Upon addition of the MPD initiator to the ΔK122 solution, the protein forms fibrils that then assemble into full length PNTs (Figures 2, 3), although the exact assembly mechanism is at this time unclear. Research in our group is on going to further characterize ΔK122-derived PNT assembly (both in solution and at surfaces), understand the structural and mechanistic requirements of PNT oligomerization and fibril stabilization, and to develop these structures for applications as bionanowires and biosensors.

## Methods

### Expression and purification of ΔK122

The truncated form of the monomeric type IV pilin from *P. aeruginosa* strain K122-4 [*pilA* (Δ1-28); ΔK122] was expressed and purified as previously reported [23,24,71,94]. The ΔK122 pilin was purified as an MBP-fusion construct and isolated from the MBP tag via cation exchange chromatography using a linear gradient of 0–1 M NaCl in 10 mM Tris (pH 7.4) following trypsin digestion of the MBP-ΔK122 fusion protein (500:1 protein:trypsin ratio; 10 min on ice). Freshly purified, monomeric ΔK122 pilin was concentrated to 15 mg·mL^-1^ or diluted to 1 mg·mL^-1^ as appropriate and used for all oligomerization experiments. All experiments conducted in this study were reviewed and approved by the York University's Biological Safety Committee, the institutional body responsible for oversight of such research.

### Determination of an optimal trigger of ΔK122 oligomerization in solution

The initially reported solution for initiating oligomerization was composed of 1.1 M 1-undecanethiol (C_11_-SH) in methanol containing 1 mM EDTA, 1 mM dithiothreitol (DTT) at pH 6.4 [23]. Studies of PNT oligomerization from surfaces suggest that the initiation of PNT assembly could be achieved with smaller chain alkylthiols, and/or mixtures thereof [24,87]. Characterization of an optimal trigger molecule in solution was conducted by incubating 15 mg·mL^-1^ ΔK122 with buffer (10 mM Tris, 300 mM NaCl, 1 mM EDTA, 1 mM DTT, pH 7.4) alone, or with buffer plus methanol (3.2 M), 1-propanol (1.7 M), 1-undecanethiol (C_11_-SH, 0.6 M), 1-tetradecanethiol (C_14_-SH, 0.5 M), 2,4-methylpentanediol (MPD, 1.0 M), polyethylene glycol 3350 (PEG 3350, 6.5% (w/v)) or polyethylene glycol 8000 (PEG 8000, 6.5% (w/v)) (Figure 1). PNT oligomerization was initiated through the addition of the trigger solution to ΔK122 in a 10:1 (v/v) protein to hydrophobe ratio, and the ΔK122-trigger solution was incubated at room temperature with nutation for 96 hours. PNT oligomerization was monitored using size exclusion chromatography (SEC) on a G50 Sephadex column (separation range 1.5 kDa - 30 kDa, standardized with blue Dextran 2000, V_0_/V_Total_ = 17.23/49.9l mL) on an Akta Purifier (GE Healthcare) at a flow rate of 1 mL·min^-1^.

### Multi-angle light scattering of ΔK122 oligomerization

The oligomerization of 1 mg·mL^-1^ ΔK122 triggered with MPD in buffer was analyzed using SEC and multi-angle light scattering (MALS) using an Akta Purifier 10 (GE Healthcare) connected in-line to a Dawn Heleos II and Optilab T-rEX light scattering system (Wyatt Technology) (Figure 2, 3). Analysis of 100 μL protein samples was performed at a flow rate of 0.5 mL min^-1^ in SEC buffer (10 mM Tris, pH 7.4) on a silica-based column (Wyatt Technology, 10 nm pore size, separation range 100 Da - 100 kDa, V_Total_ = 10.71 mL). After chromatographic separation, the column eluate traveled to the MALS flow cell where light scattering (658 nm laser light source) of the separated species was monitored by 15 angularly separated static light scattering detectors and a quasi-elastic light scattering (QELS) detector at a collection angle of 100.2° (Figure 3A; Table 1). Hydrodynamic radii (R_h_) and diffusion coefficients (D_t_) were calculated from an autocorrelation function using the accompanying Astra 6 software package (Table 1).

### Transmission electron microscopy

Transmission electron microscopy (TEM) of SEC-MALS separated PNTs was conducted in the Department of Biology’s Core Imaging Facility at York University, and samples for TEM analysis were prepared as follows. Ten microlitre aliquots of PNT solutions were dispensed onto plastic-coated nickel mesh grids and allowed to dry in air for 10 min; any remaining liquid was carefully removed by blotting with filter paper. Samples were stained with a 4% aqueous uranyl acetate, which was added to the grid and allowed to incubate for 10 minutes at room temperature, following which excess stain was removed by blotting with filter paper. Samples were imaged using a Philips 210 Transmission Electron Microscope operating at an accelerating voltage of 60 kV, and images visualized using the ImageJ software package [97].

## Abbreviations

PNTs: Protein nanotubes; ΔK122: The truncated pilin from *P. aeruginosa* strain K122-4; SEC: Size exclusion chromatography, MALS, multi-angle light scattering; TEM: Transmission electron microscopy; MPD: 2-methyl-2,4-pentanediol.

## Competing interests

The authors declare that they have no competing interests.

## Author contributions

The manuscript was written through contributions of all authors. All authors have given approval to the final version of the manuscript.
